# Anion-Exchange Membrane “Polikon A” Based on Polyester Fiber Fabric (Functionalized by Low-Temperature High-Frequency Plasma) with Oxidized Metal Nanoparticles

**DOI:** 10.3390/membranes13080742

**Published:** 2023-08-18

**Authors:** Denis Terin, Marina Kardash, Denis Ainetdinov, Timur Turaev, Ilya Sinev

**Affiliations:** 1 Yuri Gagarin State Technical University of Saratov, 77 Polytechnicheskaya Street, 410008 Saratov, Russia; terinden@mail.ru (D.T.); denis-ajjnetdinov@rambler.ru (D.A.); tim.tur.al@gmail.com (T.T.); 2 Saratov State University, 83 Astrakhanskaya Street, 410012 Saratov, Russia; sineviv@gmail.com; 3 MIREA–Russian Technological University, 78 Vernadsky Avenue, 119454 Moscow, Russia

**Keywords:** anion-exchange membrane, polyester fiber, composite heterogeneous materials, polyfunctional anion exchanger, SEM, EDX, hydrophobic/hydrophilic balance, static anion exchange capacity

## Abstract

An experimental laboratory set of samples of composite heterogeneous anion-exchange membranes was obtained by us for the development of our original method of polycondensation filling. Anion-exchange membranes were prepared on plasma-treated and non-plasma-treated polyester fiber fabrics. The fabric was treated with low-temperature argon plasma at a power of 400 W for 10 min at a pressure of 5 × 10^−5^ mbar. On the surface and bulk of the polyester fiber, a polyfunctional anionite of mixed basicity was synthesized and formed. The anion-exchange membrane contained secondary and tertiary amino groups and quaternary ammonium groups, which were obtained from polyethylene polyamines and epichlorohydrins. At the stage of the chemical synthesis of the anion matrix, oxidized nanoparticles (~1.5 wt.%) of silicon, nickel, and iron were added to the monomerization composition. The use of ion-plasma processing of fibers in combination with the introduction of oxidized nanoparticles at the synthesis stage makes it possible to influence the speed and depth of the synthesis and curing processes; this changes the formation of the surface morphology and the internal structure of the ion-exchange polymer matrix, as well as the hydrophobic/hydrophilic balance and—as a result—the different operational characteristics of anion-exchange membranes.

## 1. Introduction

**Figure 1:**
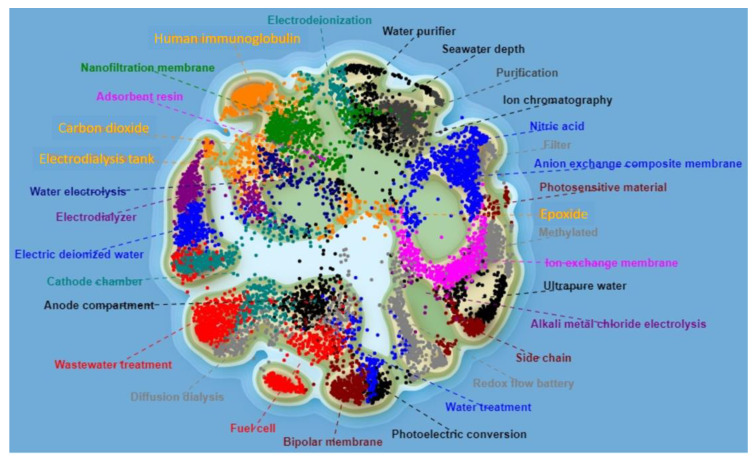
Landscape by technology clusters of patent families “anion-exchange membrane”.

Ion-exchange membranes combine the interesting properties of organic and inorganic compounds and represent an inexhaustible object of research and potential applications. The widespread introduction of methods for the purification and separation of various solutions is associated with the environmental safety and energy efficiency of many technological processes. In order to understand the scientific, research, technological, and commercial perspectives and limitations of the global market for anion-exchange membranes (AEMs), this paper assessed their patent potential in retrospect. With the help of analytical tools “Questel IP Business Intelligence Orbit Intelligence” (www.orbit.com; accessed on 21 June 2023), we have compiled a landscape of leading technology clusters and application areas for the keywords “anion-exchange membrane” and “polycondensation” identified to date (Figure 1). A patent search revealed 9137 patents containing the keyword “anion-exchange membrane” from January 1950 to June 2023. The Landscape by Technology Clusters allowed us to identify the main players—the patent family holders—as well as to gain a comprehensive view of the AEM market, including market drivers, constraints, growth opportunities, technological advances, and the micro and macroeconomic factors influencing development dynamics.

Review [1] presents basic information on research into AEMs, describes the needs of and promising directions in this area, and indicates that AEMs provide one possible route to creating fuel cells with a low content of platinum or without any platinum. It is noted that the high conductivity of hydroxide and bicarbonate anions has been demonstrated in a number of formats in AEMs, while the internal stability of membrane polymers and the demonstration of a long service life in these devices remain questionable. Membrane material strategies, detailed structure-property analysis, and insights into materials’ performance and degradation have pushed the field into the mainstream of fuel cell and energy-related device research [1,2,3,4,5,6,7,8,9,10,11]. However, most currently reported materials have not proven their usefulness in device stability tests lasting more than 1000 h [1]. For the wide application of AEMs, a high selectivity for ion transfer is important—but in most cases, there is a dependence: the higher the flux density of the target component through the membrane, the lower the selectivity of the process [2]. The key component for the electrolysis of water with an AEM is a cheap, stable, gas-tight, and good hydroxide-conducting polymer AEM. Articles [3,4,5,6,7,8,9,10,11] present target values and specifications for AEMs, discuss chemical structures and associated degradation pathways, and review the most-known and promising commercial AEMs, as well as their properties and the characteristics of the water electrolyzers that use these membranes. The authors of [5] came to the conclusion that all materials (membrane, catalyst, cell design, etc.) should be optimized together. That is, it is difficult to accurately rank commercial membranes by analyzing the available literature data, limiting the experimental ranking system.

In article [11], a composite AEM was developed and prepared via the activation of a commercial support structure with a commercially available functional group through a phase-inversion process. Zhou et al. [12] reported an original method for the separation of hydrochloric acid and oxalic acid from rare earthy oxalic acid in the processing of a starting solution using electrodialysis. Quaternary ammonium AEMs were reported in one study [13] to be more effective in diffusion dialysis, with a thin membrane being the most effective. However, their chemical stability and conductivity were still of great concern, which appears to be a major challenge for the development of AEM-based energy systems [14,15,16].

AEMs are often used in electrodialysis, pervaporation, and diffusion dialysis. The main problem associated with AEMs are their hydrophilicity, which limits the ion transport rate in the diffusion dialysis process. In order to improve the performance of AEMs used in acid recovery from industrial wastewater, this study adopted a new strategy in which brominated poly (2,6-dimethyl-1,4-phenyleneoxide; BPPO) and polyepichlorohydrin (PECH) were used as the polymer backbone of the prepared membrane. The new AEM with a net structure was formed by quaternizing BPPO/PECH with N,N,N,N-tetramethyl-1,6-hexanediamine [17]. It was shown in [18] that, in addition to steric hindrances, the transfer of phosphates in AEMs are affected by the ion-exchange capacity and the composition of fixed groups (strongly basic, weakly basic), as well as the degree of crosslinking of the polymer matrix. In spite of the wide variety of commercial AEMs, their characteristics—in particular their electrical conductivity and counterion permselectivity—are unsatisfactory for some applications, such as electrolyte solution concentration [19]. Poly(alkyl-biphenyl pyridinium)-based AEMs with alkyl side chains were synthesized for permselective anion separation [20]. By altering the length of the grafted side chain, the hydrophilicity and other attributes of the membranes could be controlled. Tetrakis(dialkylamino)phosphonium (TKDAAP) compounds exhibit extraordinary base resistance—a prerequisite feature for high-performance AEMs [21]. For AEM water electrolysis, two types of AEMs containing crosslinked poly(phenylene oxide) and poly(styrene ethylene butylene styrene) were prepared with and without triazole [22]. In [23], a new approach is provided for the improvement of ion conductivity at a low ion content by introducing perfluorinated branch chains—putting forward a standardized method for the preparation of AEMs with high performance. In the study [24], two different types of ion-exchange membranes were used to investigate the tendency of membrane fouling with respect to surface roughness and hydrophilicity. The membranes with a rough surface showed a higher fouling tendency than those with a smooth surface in the short-term continuous fouling tests [24]. During the cyclic operations of fouling and mitigation of the commercially available membranes, the irregularities of the rough membrane surface caused a rapid increase in electrical resistance from the beginning of the fouling due to excessive adsorption on the surface; however, the fouling was easily mitigated by the hydrophilic surface.

It is known that the use of microwave radiation at various stages in the obtaining of unsaturated polyester composite materials, which are obtained in the presence of modified carbon nanotubes, affects the processes of the structural formation of composites [25]. The work in [26] presents a study of the possibility of directly controlling the performance properties of epoxy composites. Futhermore, this possibility occurs when adding small concentrations of thermally expanded graphite–graphene structures; as a result, a number of the operational characteristics of the produced materials improve (thermal, fire, and heat resistance, as well as the thermal conductivity coefficient) [27]. Modification of the epoxy polymer with the presented plasticizers affects the carbonization process [28]. The possibility of using low concentrations of ultrafine polytitanates as fillers [29] has been studied; the results showed that it possible to control the processes of structural formation and the properties of the epoxy composite in this manner [30].

As rightly noted in [31,32,33], chemical transformations initiated by plasma in a thin surface layer of a polymer (including polyester fibers) cause changes in the structure of this layer—its electrical, physical, mechanical, optical, sorption, and other properties—which ultimately leads to the desired technological effects.

Comprehensive studies of the influence of the method of manufacturing a fibrous base on the properties of ion-exchange membranes have been carried out by the authors of articles [34,35,36,37,38,39]. In these studies, polyester fibers pretreated with low-temperature high-frequency plasma were proposed as the fibrous substrate of the membranes. In addition, the possibility was considered of doping this base with ultrafine oxides of silicon, iron, and nickel.

This study continues this series of investigations; it focuses on the effects of the low-temperature plasma treatment of fibers and the introduction of nanoparticles of silicon, iron, and nickel oxides on the hydrophilic/hydrophobic balance of the surface of the textile structure of the fibrous base of anion-exchange membranes, as well as their ion-exchange capacity.

## 2. Materials and Methods

### 2.1. Membrane Fabrication

We fabricated laboratory pilot-scale batches of membranes “Polikon A” (letter “A” denotes an AEM, and the term “Polikon” corresponds to the original technological method of membrane production fabricated by the authors [34,35,36,37,38,39]). Membranes were formed using polyester fiber-based fabrics treated and not treated with plasma.

A feature of this work was the preliminary basic pretreatment of a fabric based on a polyester fiber under low-temperature ion-plasma conditions. The treatment was carried out with low-temperature HF argon plasma at a power of 400 W for 10 min at a pressure of 5× 10^−5^ mbar (compact high-vacuum installation MTI VTC-600-PVD (Republic of Korea)).

“Polikon A” membranes were obtained by polycondensation filling of a fibrous matrix with a monomerization composition and further formation of an anion-exchange matrix, both on the surface of the fibrous base of the fiber and in its structure [26,27,28,29]. The essence of the method of polycondensation filling is to carry out the synthesis of a polymer binder directly on the surface and in the bulk of the fiber, which can significantly improve the performance of the composite material. Indeed, the introduction of chemical fibers with reactive groups in their structure into the monomer at the synthesis stage facilitates the access of monomers to these groups, while the interaction of the filler and the monomer is possible with the formation of a special structure of a composite material with specific properties [37].

The technological process consisted of the pretreatment of the raw materials, the preparation of monomerization composites (on the basis of polyethylene polyamines and epichlorohydrins), and their application on a fibrous base; then, the synthesis and curing process was carried out, thereby forming a spatial network of polymer matrices on the surface and in the volume of the system [34,35].

The ratio of the polymer matrix to the fibrous base in the finished membrane was 60 to 40 mass %. The ratio of 60% polymer matrix and 40% fibrous filler was strictly (exactly) maintained using a number of techniques. This ratio did not depend on the volume of the fabricated batch of material (in absolute terms, about several tens of kilograms).

### 2.2. Materials

For the manufacture of the anion-exchange membrane “Polikon A”, the following materials were used: “Lavsan fabric” (filter cloth FL-4 (Russia), SKU C-217, GOST 26095-84 Polyester industrial filter fabrics. Specifications), polyethylene polyamine (Russia, Specifications—TU 2413-357-00203447-99), and epichlorohydrin (Russia, GOST 12844-74 Ion-exchange resins. Anionites. Specification).

In this study, we used ultrafine colloidal silicon oxide with a predominant amorphous phase and a specific surface area (SSA) of ~300 m^2^/g, and ultrafine oxides of nickel (SSA~9 m^2^/g) and iron (SSA~18 m^2^/g) obtained by plasma technology via the recondensation of ultrafine materials in the laboratory of the State Scientific Research Institute of Chemistry and Technology of Organoelement Compounds (GNIIChTEOS) [40]. Oxidized nanoparticles were added to the monomerization composition (no more than 1.5 wt.%) at the stage of membrane formation.

### 2.3. Methods for Studying Properties

The morphology and thicknesses of the samples were determined on an analytical complex based on a MIRA 2LMU scanning electron microscope. The microanalysis was carried out by the energy dispersive method using the INCA Energy 350 system. Under the chosen modes of measurements and the position of the sample with respect to the electron beam, the depth of generation of the characteristic X-ray radiation was 1 μm.

To study the surface hydrophilic/hydrophobic balance, a precision optical tensiometer Theta Lite Optical Tensiometer TL 100 was used [41]. A drop of distilled water with a volume of 14.58 mL was applied to the surface. The droplet shape was recorded in the “Fast + Normal” modes. The “Fast” mode corresponds to 50 frames with an intensity of 16 ms. The “Normal” mode corresponds to 50 frames with an intensity of 1 s. The obtained video images were processed using a specialized AttensionThetta computer program, which allowed the obtaining of an approximation of the experimental curves—using the Young–Laplace equation [41,42]—to calculate the contact angles with an error of ±0.1 degrees in the range of 0 to 180 degrees.

The static ion-exchange capacity (E_n_, mg eq/g) was determined using an acid–alkali method. A portion of the crushed membrane was converted into the hydroxyl form and placed in a given volume of 0.1 M HCl for 24 h. The concentration of HCl in this solution was determined by titration with 0.1 M NaOH. The static ion exchange capacity was calculated by the loss of acid from the equilibrium solution.

## 3. Results

**Figure 2:**
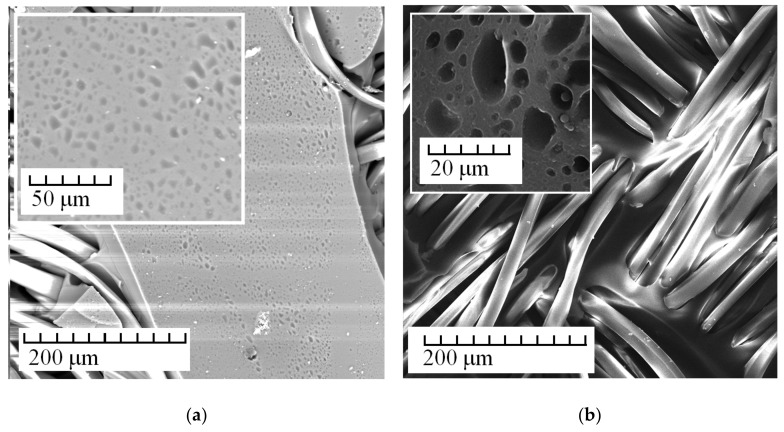
SEM images of Polikon A on untreated (**a**) and plasma-treated (**b**) polyester fiber.

**Figure 3:**
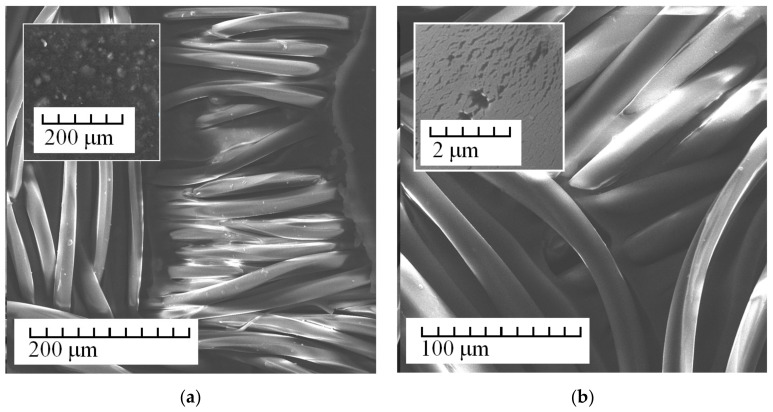
SEM images of Polikon A on untreated (**a**) and plasma-treated (**b**) polyester fiber, with the addition of ultra-dispersed silicon oxide.

**Figure 4:**
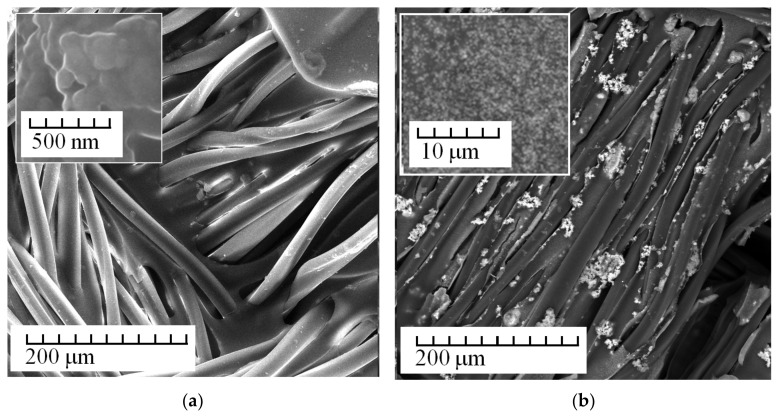
SEM images of Polikon A on untreated (**a**) and plasma-treated (**b**) polyester fiber, with the addition of ultrafine iron oxide.

**Figure 5:**
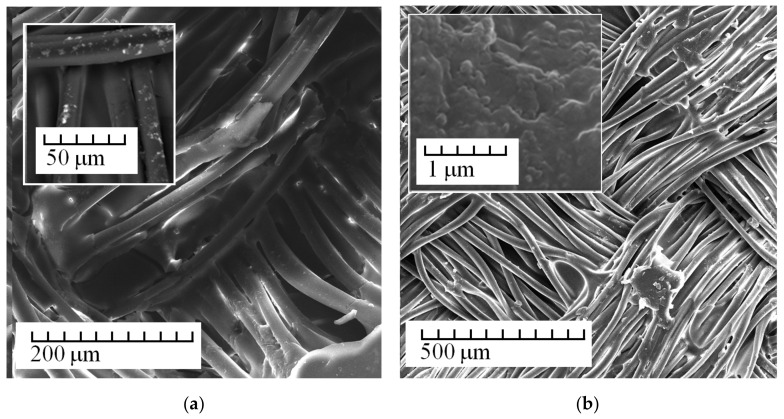
SEM images of Polikon A on untreated (**a**) and plasma-treated (**b**) polyester fiber, with the addition of ultrafine nickel oxide.

Figure 2, Figure 3, Figure 4 and Figure 5 show the most typical SEM images of the obtained membranes. Figure 2a presents images of membranes obtained using tissue not treated with plasma. Figure 2b shows treated tissue. Figure 2 (on the insets) shows the area of a smooth transition between the fiber and polymer matrix and typical images of a porous anion-exchange matrix. It follows from the images that plasma treatment leads to the ordering of the system of macropores and channels.

Figure 3 shows typical images of the surface of Polikon A membranes with the addition of colloidal silicon oxide. The inset shows a region of smooth transition between the fiber and the polymer matrix and the distribution of silicon oxide in the polymer matrix.

No noticeable system of hierarchical macropores was found in the samples not treated with plasma. By contrast, on the samples treated with plasma a branched system of cylindrical macropores was revealed, as well as characteristic areas observed as having non-through-ordered channels, or wrinkles.

It was found that the highest concentration of silicon oxide was evenly distributed over the polymer matrix. Areas with agglomerates of silicon oxide nanoparticles (in Figure 3a) may have been insignificantly present.

Figure 4 shows a typical morphology of Polikon A membranes with iron oxide nanoparticles.

The polymer matrix had a smoothed landscape structure with insignificant height transitions (samples not treated in plasma, Figure 4a). In contrast to the case in Figure 3, the iron oxide nanoparticles (Figure 4) were more evenly distributed over the monomerization composition (see photo inset, Figure 4b) and were able to agglomerate on the polyester fabric fibers (on plasma-treated samples).

Figure 5 shows the surface morphology of Polikon A membranes with nickel oxide nanoparticles. It was observed that the nickel oxide nanoparticles were generally uniformly agglomerated on the polyester fibers of the fabric.

**Figure 6:**
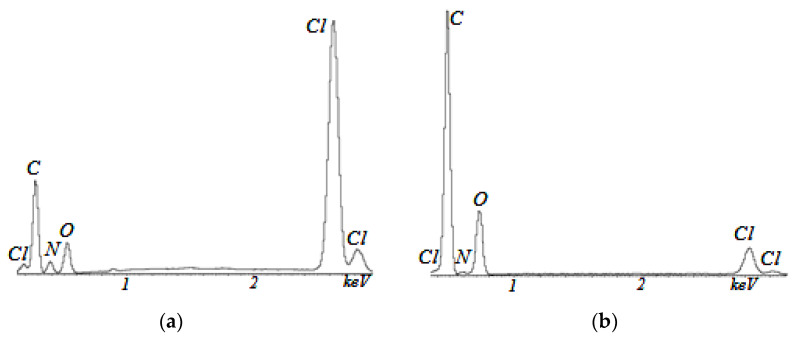
EDX analysis of Polikon A with untreated (**a**) and plasma-treated (**b**) polyester fiber.

**Figure 7:**
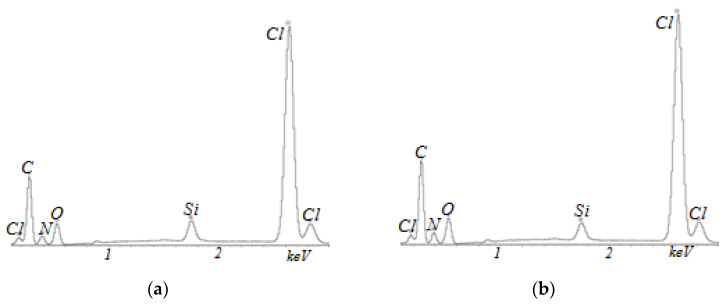
EDX analysis of Polikon A with untreated (**a**) and plasma-treated (**b**) polyester fiber and the addition of ultra-dispersed silicon oxide.

**Figure 8:**
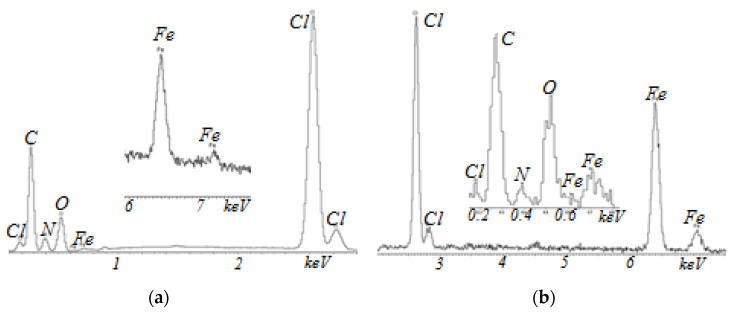
EDX analysis of Polikon A with untreated (**a**) and plasma-treated (**b**) polyester fiber and the addition of ultrafine iron oxide.

**Figure 9:**
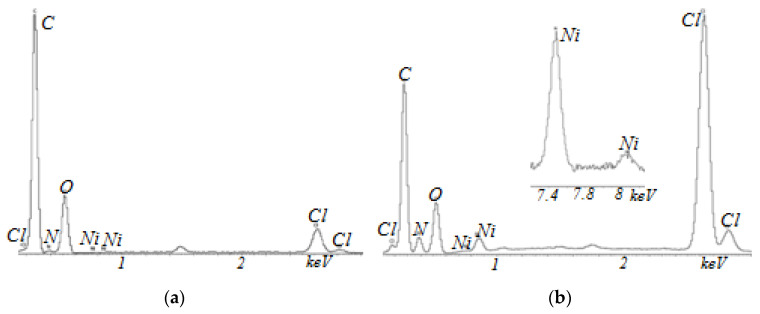
EDX analysis of Polikon A with untreated (**a**) and plasma-treated (**b**) polyester fiber and the addition of ultrafine nickel oxide.

The chemical composition of Polikon A on the fabric from the polyester fiber was studied by the energy dispersive method (Figure 6, Figure 7, Figure 8 and Figure 9). It was shown that the elemental composition was dominated by carbon (in weight%): 49.96 ± 0.37; nitrogen 17.17 ± 0.43; chlorine 16.61 ± 0.13; and oxygen 16.26 ± 0.22 (Figure 6) in the untreated polyester fabric. Plasma treatment in the fabric made of polyester fibers changed the balance of the content of elements: carbon 61.56 ± 0.52; oxygen 32.88 ± 0.38; nitrogen 3.20 ± 0.67; and chlorine 2.33 ± 0.04.

Suffusion is a destructive process that occurs during the operation of membranes, expressed in the leeching of unrelated or loosely bound particles from the material into the solution [34]. To avoid suffusion, as well as to give the anion matrix “Polikon A” a more homogeneous cross-linked structure, it was proposed to introduce the oxidized ultrafine modifying additives of Fe, Ni, and Si oxides at the synthesis stage [34].

For samples with the addition of silicon oxide nanoparticles, ultra-dispersed iron oxide, and nickel oxide, the balance of the content of elements was practically unchanged (Figure 7, Figure 8 and Figure 9). The main changes appeared in the oxygen–nitrogen ratio—namely, the presence of oxygen increased in samples treated with plasma, and the nitrogen content decreased.

**Figure 10:**
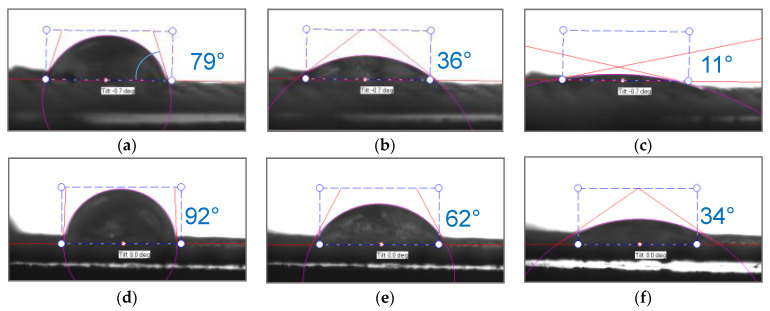
Optical microscopy of dynamic contact angle for Polikon A with untreated (**a**–**c**) and plasma-treated (**d**–**f**) polyester fiber: 0.050 s (**a**,**d**), 0.544 s (**b**,**e**), 1.836 s (**c**,**f**).

**Figure 11:**
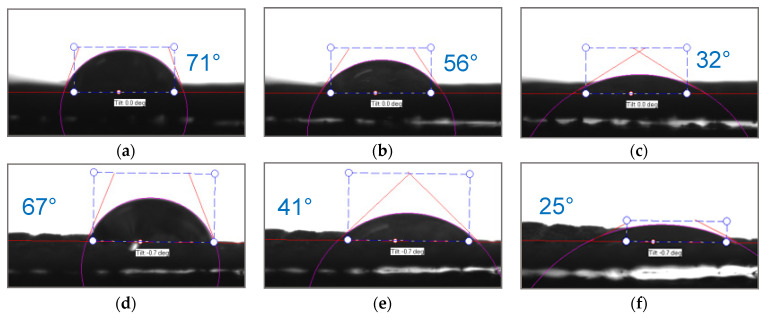
Optical microscopy of dynamic contact angle for Polikon A with untreated (**a**–**c**) and plasma-treated (**d**–**f**) polyester fiber, with the addition of ultra-dispersed silicon oxide: 0.050 s (**a**,**d**), 0.544 s (**b**,**e**), 1.836 s (**c**,**f**).

**Figure 12:**
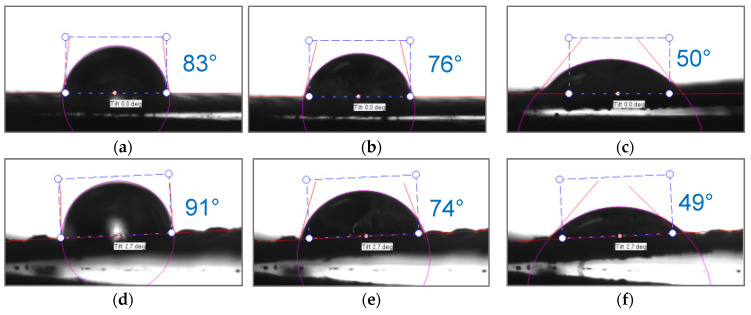
Optical microscopy of dynamic contact angle for Polikon A with untreated (**a**–**c**) and plasma-treated (**d**–**f**) polyester fiber, with the addition of ultrafine iron oxide: 0.050 s (**a**,**d**), 0.544 s (**b**,**e**), 1.836 s (**c**,**f**).

**Figure 13:**
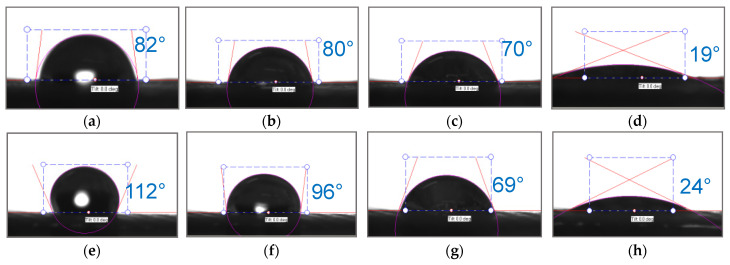
Optical microscopy of dynamic contact angle for Polikon A with untreated (**a**–**c**) and plasma-treated (**d**–**f**) polyester fiber, with the addition of ultrafine nickel oxide: 0.050 s (**a**,**e**), 0.544 s (**b**,**f**), 1.836 s (**c**,**g**), 11.835 s (**d**,**h**).

The hydrophilic/hydrophobic balance of the surfaces of the fabric made of polyester fibers (before and after treatment) and the heterogeneous anion-exchange material Polikon A was studied. Micrographs of drops are presented on a single scale (Figure 10, Figure 11, Figure 12 and Figure 13).

**Figure 14:**
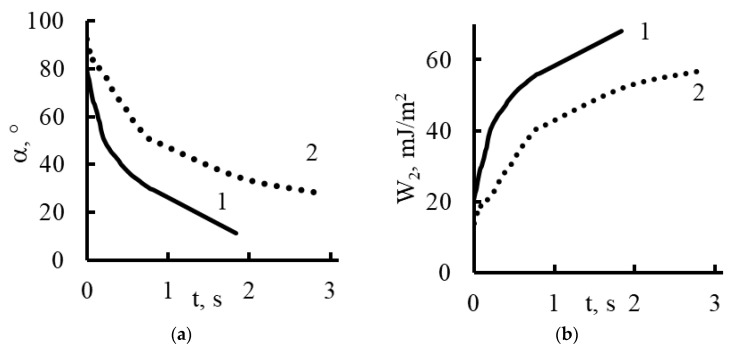
Dynamic contact angle (**a**) and work of adhesion (**b**) for Polikon A with untreated (curve 1) and plasma-treated polyester fiber (curve 2).

As is known, the nature of adhesion that occurs when wetting solid rough surfaces with water is due to hydrogen bonds and dispersion interactions. The latter is decisive for adhesive interaction on surfaces with a changing hydrophobic/hydrophilic balance. For plasma-treated membranes, the contact angle becomes higher and their rate of change decreases (Figure 10 and Figure 14).

Plasma treatment shifts the hydrophobic/hydrophilic balance of the surface to a relatively greater hydrophobicity, and this trend is maintained until complete wetting is achieved.

**Figure 15:**
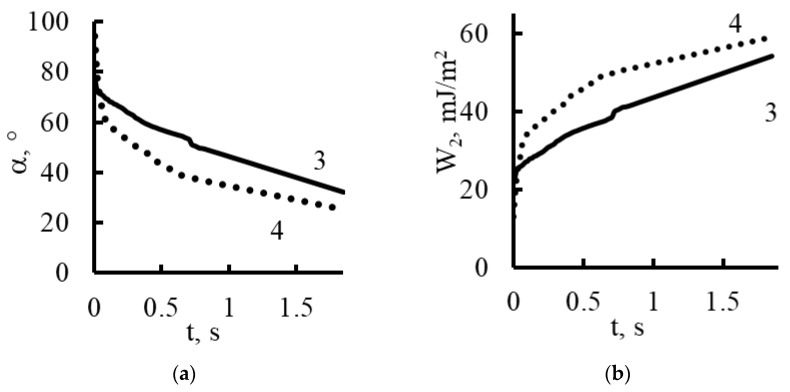
Dynamic contact angle (**a**) and work of adhesion (**b**) for Polikon A with untreated (curve 3) and plasma-treated polyester fiber (curve 4) and the addition of ultra-dispersed silicon oxide.

When silicon oxide nanoparticles were introduced (Figure 11 and Figure 15), the contact angles for the plasma-treated and untreated samples were 94° and 77°, respectively, at the moment the drop touched the surface of the membrane. After 0.03 s, these angles were compared, and then the processes for reducing the contact angle proceeded more smoothly in the case of the untreated membranes. The contact angle on the plasma-treated membranes with the silicon oxide nanoparticles gradually decreased to 25° until complete wetting of the sample was reached.

**Figure 16:**
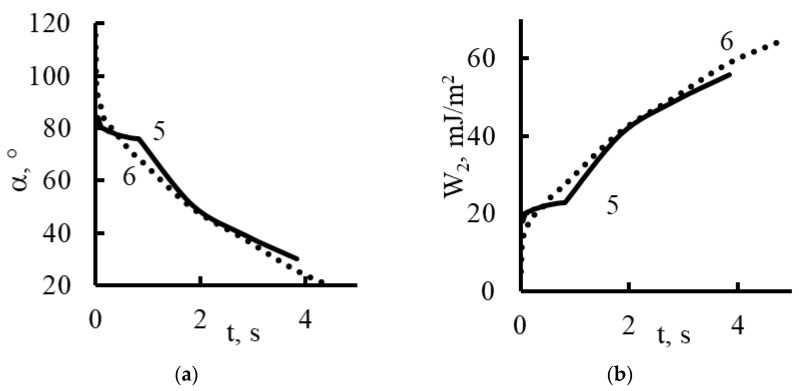
Dynamic contact angle (**a**) and work of adhesion (**b**) for Polikon A with untreated (curve 5) and plasma-treated polyester fiber (curve 6) and the addition of ultrafine iron oxide.

When iron oxide nanoparticles were introduced (Figure 12 and Figure 16), the contact angle for the plasma treatment membranes was about 115° at the moment the drop touched the surface of the membrane. In the case of the sample without treatment, the contact angle was equal to about 80°. After 0.5 s, the values of the contact angles were compared, and they were practically unchanged until the completion of the wetting process.

**Figure 17:**
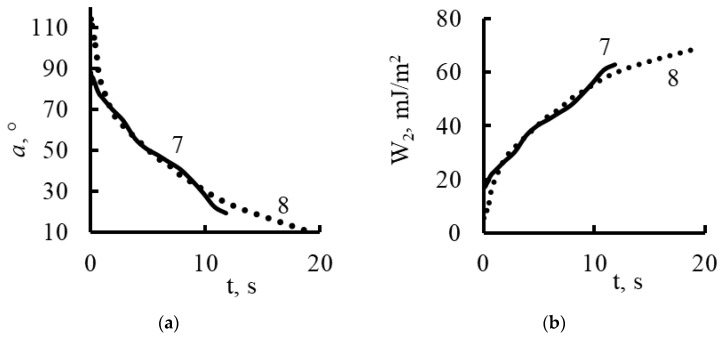
Dynamic contact angle (**a**) and work of adhesion (**b**) for Polikon A with untreated (curve 7) and plasma-treated polyester fiber (curve 8) and the addition of ultrafine nickel.

Nickel oxide nanoparticles (Figure 13 and Figure 17) had a significant effect on the hydrophobic/hydrophilic balance of the surface of both the untreated and plasma-treated membranes. The process of complete wetting was the longest for these samples.

The contact angle for the plasma treated and untreated membranes was about 114^o^ and 88°at the moment the drop touched the surface. The contact angle values became comparable and equal to 71° only after 1.8 s. Then, within 12 s, there was a smooth change in the values of the contact angle up to 20° for the untreated membranes, and within 18 s to 10° for the membranes treated with plasma.

Using the contact angle values (α), we determined the work of adhesion (*W*_2_) from the equation [42,43]:

$$W_{2}^{}=\sigma_{lg}\left[1-{\left(\frac{2-3\cos\alpha +cos^{3}\alpha }{4}\right)}^{\frac{1}{3}}\right]$$

Here, the surface tension of water *σ_lg_* is equal to 72.7 mJ/m^2^. The results of the calculations are shown in Figure 14, Figure 15, Figure 16 and Figure 17.

Based on the fact that during the spreading of a drop, the liquid–gas interface changes much less than the liquid–membrane interface, we believe that the work of adhesion calculated per unit area of the surface of a spherical drop that comes into contact with the gas medium is a more complete characterization of the adhesion work and a better method for calculating the area of liquid contact with the membrane surface per 1 cm^2^ [43].

As follows from the graphs in Figure 14, Figure 15, Figure 16 and Figure 17, the work of adhesion increased with decreases in the contact angle—this was true for both the untreated and plasma-treated membranes; the incorporation of nanoparticles also did not change this characteristic.

The value of the adhesion work on the membrane samples treated with plasma (Figure 14) was significantly lower than for the untreated ones, which indicates a change in the nature of the hydrophobic/hydrophilic balance of the surface.

The adhesive stress (lines characterizing the directions of the surface tension of the membrane) on the hydrophobic areas of the surface prevented the drop from spreading, and on the hydrophilic areas, it contributed to the drop spreading.

In the presence of silicon oxide nanoparticles, the work of adhesion in the plasma-treated membrane was greater than that in the untreated one, which indicates a significant predominance of surface hydrophilization.

The increase in the work of adhesion can be explained by the fact that the adhesion of membranes containing silicon oxide is determined not only by dispersion interactions, but also by hydrogen bonding.

The surface of the membranes included oxygen atoms of siloxane groups -Si-O-Si-, which have valence bonds with silicon atoms.

Hydrogen bonding of the membrane surface with water is due to the fact that the oxygen atom of the siloxane groups has two free pairs of electrons that can interact with the hydrogens of the water molecule.

The incorporation of nanoparticles of iron oxide (Figure 16) and nickel oxide (Figure 17) smoothed the effects of the plasma treatment; the adhesion work values changed monotonically and had almost the same values.

The incorporation of nanoparticles of iron oxide and nickel oxide shifted the hydrophobic/hydrophilic balance of the membrane surface to the region of relative hydrophobicity.

The time for complete wetting increased significantly; this was especially noticeable in the case of the incorporation of nickel oxide nanoparticles into the plasma-treated membrane.

Table 1 examines the complex effects of the low-temperature ion-plasma pretreatment of fabrics made of polyester fibers, as well as the effects of embedded metal oxide nanoparticles on the sorption characteristics of the anion-exchange membrane material Polikon A.

We determined the static anion exchange capacity of the resulting membranes; it was found that the incorporation of nanoparticles at the stage of membrane formation led to an increase in the static exchange capacity in the case of silicon oxide by 6.2%, iron oxide by 17%, and nickel oxide by ~56%.

Plasma treatment of the polyester fiber fabric reinforced the trend and led to a 44% increase in static exchange capacity.

The combined effect of plasma treatment and the incorporation of oxide nanoparticles led to an increase in the static exchange capacity by 57% (silicon oxide), 36% (iron oxide), and 83% (nickel oxide).

It was noted that nanoparticles (which we introduced into the monomerization composition) formed a more branched and accessible structure for ion exchange, while the availability of ion-exchange groups attached to the polymer framework increased. After plasma treatment of the polyester fiber (fabric), a long-term effect of increasing the wettability of the fiber—improving its capillarity and sorption—was observed, which must certainly affect the processes of synthesis and curing of the anion-exchange matrix, since the polyester fiber itself plays the role of nanoreactors. Our research has shown that plasma treatment affects the whole picture of ion exchange in general. Thus, based on the data in Table 1 and the study of the hydrophobic/hydrophilic balance of the membrane surface (Figure 14, Figure 15, Figure 16 and Figure 17), we can speak of a significant effect of processing fabrics based on polyester fibers with low-temperature high-frequency plasma on the sorption characteristics of AEM materials. The AEM Polikon A obtained by us is promising for use in units for the capacitive deionization of aqueous solutions [44].

## 4. Conclusions

In this work, we have demonstrated the effects of the low-temperature plasma treatment of fabrics based on polyester fibers for the subsequent controlled polycondensation synthesis of anion-exchange composite heterogeneous membranes.

The membranes were obtained using polyester fibers treated and not treated with low-temperature argon plasma at a power of 400 W for 10 min and at a pressure of 5 × 10^−5^ mbar. A polyfunctional anion exchanger of mixed basicity was formed on the surface and in the bulk of the polyester fiber. The AEM contained secondary and tertiary amino groups and quaternary ammonium groups, which were obtained from polyethylene polyamines and epichlorohydrins. At the stage of formation of the AEMs, oxidized nanoparticles (~1.5 wt.%) of silicon, nickel, and iron were added to the monomerization composition.

For samples doped by nanoparticles of silicon oxide, ultrafine iron oxide, and nickel oxide, the balance of the element content remained practically unchanged. The main changes were manifested in the oxygen–nitrogen ratio. The presence of oxygen increased in samples treated with plasma, while the nitrogen content decreased.

The treatment of the surface of the polyester fiber with low-temperature plasma led to a change in the hydrophobic/hydrophilic balance of the polyester fibers in the fabric, as well as the capillarity, moisture sorption, and adhesive properties of the fibrous base. The work of adhesion increased with decreasing contact angles in the case of the untreated and plasma-treated membranes. The introduction of nanoparticles did not change this trend. Plasma treatment shifted the hydrophobic/hydrophilic balance of the surface to a relatively greater hydrophobicity. In the presence of silicon oxide nanoparticles, the work of adhesion for the plasma-treated membrane was greater than for the untreated one. This fact indicates a significant predominance of surface hydrophilization. The incorporation of iron oxide and nickel oxide nanoparticles shifted the hydrophobic/hydrophilic balance of the membrane surface to relative hydrophobicity. In addition, the plasma treatment of the polyester fiber fabric led to an increase of almost 20% in the static ion-exchange capacity of the membranes. The synergistic effects of plasma treatment and the incorporation of oxide nanoparticles reinforced this trend. The greatest increase in the ion-exchange capacity (by 80%) took place when the polyester fibers of the membrane were treated with low-temperature plasma and doped with nickel oxide nanoparticles.

We believe that these new membranes are promising for use in the capacitive deionization of various solutions.

## Figures and Tables

**Table 1:** Static ion-exchange capacity of Polikon A membranes.

Polikon A	Ion-Exchange Capacity, mg-eq/g	
	without Plasma Treatment	with Plasma Treatment
without nanoparticles	2.60 ± 0.05	3.12 ± 0.05
Si oxidized nanoparticles	2.76 ± 0.05	4.09 ± 0.05
Fe oxidized nanoparticles	3.05 ± 0.05	3.53 ± 0.05
Ni oxidized nanoparticles	4.07 ± 0.05	4.73 ± 0.05

## Data Availability

Not applicable.
